# Detecting Diverse Seizure Types with Wrist-Worn Wearable Devices: A Comparison of Machine Learning Approaches

**DOI:** 10.3390/s25175562

**Published:** 2025-09-06

**Authors:** Louis Faust, Jie Cui, Camille Knepper, Mona Nasseri, Gregory Worrell, Benjamin H. Brinkmann

**Affiliations:** 1 Robert D. and Patricia E. Kern Center for the Science of Health Care Delivery, Mayo Clinic, Rochester, MN 55905, USA; 2 Brain Neurology and Engineering Laboratory, Department of Neurology, Mayo Clinic, Rochester, MN 55905, USA

**Keywords:** seizure monitoring, wearable devices, machine learning, epilepsy

## Abstract

**Objective**: To evaluate the feasibility and effectiveness of wrist-worn wearable devices combined with machine learning (ML) approaches for detecting a diverse array of seizure types beyond generalized tonic–clonic (GTC), including focal, generalized, and subclinical seizures. **Materials and Methods**: Twenty-eight patients undergoing inpatient video-EEG monitoring at Mayo Clinic were concurrently monitored using Empatica E4 wrist-worn devices. These devices captured accelerometry, blood volume pulse, electrodermal activity, skin temperature, and heart rate. Seizures were annotated by neurologists. The data were preprocessed to experiment with various segment lengths (10 s and 60 s) and multiple feature sets. Three ML strategies, XGBoost, deep learning models (LSTM, CNN, Transformer), and ROCKET, were evaluated using leave-one-patient-out cross-validation. Performance was assessed using area under the receiver operating characteristic curve (AUROC), seizure-wise recall (SW-Recall), and false alarms per hour (FA/h). **Results**: Detection performance varied by seizure type and model. GTC seizures were detected most reliably (AUROC = 0.86, SW-Recall = 0.81, FA/h = 3.03). Hyperkinetic and tonic seizures showed high SW-Recall but also high FA/h. Subclinical and aware-dyscognitive seizures exhibited the lowest SW-Recall and highest FA/h. MultiROCKET and XGBoost performed best overall, though no single model was optimal for all seizure types. Longer segments (60 s) generally reduced FA/h. Feature set effectiveness varied, with multi-biosignal sets improving performance across seizure types. **Conclusions**: Wrist-worn wearables combined with ML can extend seizure detection beyond GTC seizures, though performance remains limited for non-motor types. Optimizing model selection, feature sets, and segment lengths, and minimizing false alarms, are key to clinical utility and real-world adoption.

## 1. Introduction

Epilepsy, a serious neurological disorder affecting over 70 million people worldwide, is characterized by recurrent seizures that disrupt muscle control, behavior, sensation, and/or consciousness [1]. Despite advancements in treatment and management strategies, epilepsy remains significantly associated with morbidity and mortality [2]. A central challenge in epilepsy management is accurate seizure monitoring, which is essential for evaluating seizure burden, recurrence risk, and treatment response [3].

Although video-electroencephalography (vEEG) remains the gold standard for seizure detection, it requires costly and time-consuming in-clinic monitoring [4]. Outside the clinic, seizure tracking has primarily relied on self-reported diaries, which are prone to under-reporting due to missed, unrecognized, amnestic, or nocturnal seizures [5,6].

To address these challenges, wearables offer an objective, non-invasive alternative for long-term, remote seizure monitoring [7]. When paired with machine learning (ML), biosignals collected by these devices have been successfully used to automate seizure detection and classification [8,9]. While previous research has focused on generalized tonic–clonic (GTC) seizures, there remains a need to expand detection capabilities to a broader range of seizure types [3,10].

Towards this aim, our study evaluated the effectiveness of wrist-worn wearables, in combination with machine learning (ML), for detecting focal, generalized, and subclinical seizures. Focal seizures originate in a specific region of the brain and can produce a wide range of symptoms depending on the affected area. We focused on two focal subtypes: focal aware-dyscognitive seizures, which involve impaired awareness and may present with confusion or automatisms; and focal hyperkinetic seizures, characterized by vigorous, often repetitive motor activity involving the limbs or trunk. In contrast, generalized seizures engage both hemispheres of the brain from onset and typically result in widespread motor manifestations. The generalized subtypes included in our analysis were as follows: generalized tonic–clonic (GTC) seizures, which involve an initial phase of muscle stiffening (tonic) followed by rhythmic jerking (clonic); myoclonic seizures, marked by brief, shock-like muscle jerks; and tonic seizures, which cause sudden muscle stiffening that can lead to falls. Subclinical seizures, on the other hand, are detected as abnormal electrical activity on an EEG but lack overt physical symptoms. Despite their subtlety, they can still have significant neurological or cognitive impacts that are difficult to detect without continuous neurophysiological monitoring. To address this challenge, we systematically compared multiple ML models, feature sets, and segment lengths to determine the most effective detection strategies for each seizure type, and to assess whether a single, unified approach could perform well across all categories.

## 2. Materials and Methods

### 2.1. Study Design

This study evaluated the efficacy of wrist-worn wearables for detecting six seizure types: aware-dyscognitive, hyperkinetic, GTC, myoclonic, tonic, and subclinical. Data were collected using the Empatica E4 (Empatica, Boston, MA, USA), a research-grade, wrist-worn device that records accelerometry (ACC); blood volume pulse (BVP, a modified measure of photoplethysmography); electrodermal activity (EDA); skin temperature; and heart rate (HR), estimated from BVP [7]. The sampling rates were 32 Hz for ACC, 64 Hz for BVP, 4 Hz for EDA, 4 Hz for skin temperature, and 1 Hz for HR [10].

Participants were recruited from Mayo Clinic, Rochester, if they were undergoing invasive video-EEG (vEEG) or admitted to the epilepsy monitoring unit for vEEG with a high clinical suspicion of seizures. A vEEG procedure combines continuous EEG recordings with synchronized video to monitor and capture the electrical and behavioral manifestations of seizure activity. The E4 device was placed on the wrist most involved during seizures, unless clinical constraints (e.g., IV tubes) prevented preferred placement, with devices positioned to avoid disrupting care. Patients were monitored simultaneously with the E4 and vEEG for an average of two to five days. To maintain continuous recording, E4s, capable of over 36 h of operation per charge, were swapped for fully charged units as needed [11].

Seizure events were identified clinically by board-certified neurologists reviewing EEG recordings with synchronized video, using clinical criteria [12] in an in-patient hospital environment as part of the patients’ presurgical epilepsy evaluation. Seizure onsets were determined based on the first significant clinical or electrographic change from baseline observed for each seizure. Most patients were monitored with a 32-channel standard scalp EEG electrode array in the standard 10-20 system montage [13]. Some patients in the cohort were monitored with a high-density 76-channel scalp electrode array placed using the international 10-10 system, and some patients had seizures monitored with EEG recorded by invasive stereotactic or subdural electrodes [14,15].

All data preprocessing and analysis were performed using Python (v3.12.1).

### 2.2. Ethics

This observational study was approved by the Mayo Clinic’s IRB after a full board review under protocol number 22-006702. All patients provided written informed consent prior to taking part in the study.

### 2.3. Data Pre-Processing and Feature Extraction

Raw biosignals from the E4s underwent multiple preprocessing steps, including signal alignment, feature extraction, and dataset partitioning.

All biosignals were resampled to 32 Hz. Linear interpolation was used for upsampling and averaging for downsampling. To prevent interpolation across non-wear periods, interpolation was restricted to consecutive wear-time blocks with no more than 60 s of missing data. Non-wear periods were identified using skin temperature readings outside the physiologically plausible range (<27 °C or >45 °C) [16]. Additional derived signals included phasic and tonic components of EDA [17] and sleep–wake states inferred from ACC [18].

The resulting multivariate time series were segmented into uniform lengths as inputs to the ML models. To assess the impact of segment length, two lengths were evaluated: 10 s and 60 s. Two data formats, *wide* and *long*, were constructed for each segment length to accommodate model-specific input specifications. In the *wide* format, each input was a feature vector derived from a full segment. Frequency-domain features were computed for each segment using the Fourier transform, including peak frequency, spectral entropy, spectral centroid, spectral bandwidth, and total power [19,20]. Within each segment, the mean value of each biosignal was also included. The *long* format retained the full time series of each segment, along with the same spectral features, extracted using the short-time Fourier transform, with a sliding window equal to half the segment length [21].

Ictal labels were assigned based on expert annotations of seizure onset and offset using vEEG. Seizures shorter than 60 s were excluded. To address class imbalance between ictal and interictal segments, ictal segments were upsampled using overlapping rolling windows, with 1 s overlap for 10 s segments and 10 s overlap for 60 s segments. This approach both mitigated class imbalance and preserved predictive signals that might otherwise have been split across non-overlapping segments. Interictal segments were downsampled to match the number of ictal segments. These sampling strategies were applied only to the training sets; the validation sets used all unique, non-overlapping segments to better reflect real-world performance.

Finally, all features underwent within-patient standardization by subtracting the mean and dividing by the standard deviation.

### 2.4. Machine Learning Task

Seizure detection was framed as a time-series classification task, where each input segment was classified as either *ictal* or *interictal*. To investigate whether certain ML approaches were better suited for specific seizure types, we evaluated three distinct modeling strategies: (1) Extreme Gradient Boosting (XGBoost), (2) deep learning architectures, including LSTMs, CNNs, and Transformers, and (3) Random Convolutional Kernel Transform (ROCKET), along with its revisions MiniROCKET and MultiROCKET [22,23,24,25,26,27,28]. The XGBoost models were trained on the *wide* formats, while the deep learning and ROCKET models were trained on the *long* formats. XGBoost and deep learning models have previously demonstrated strong performance in seizure detection and forecasting, while ROCKET has shown broad effectiveness in time-series classification tasks [29,30].

To ensure robust model evaluation, we tested multiple hyperparameter settings. XGBoost varied in estimators, depth, and learning rate; deep learning models in size, layers, and dropout; and ROCKET variants in kernel count. Full details are provided in Supplementary Materials, Section S1. Model evaluation was conducted using leave-one-patient-out cross-validation, treating each patient as an independent fold to mitigate overfitting and assess generalizability.

To evaluate the relative importance of different biosignals and explore the feasibility of seizure detection across varying device configurations, four distinct feature sets were constructed. The **ACC set** assessed the predictive power of ACC alone. Comparable performance between this set and more complex ones would suggest that seizure detection may be equally feasible using only an accelerometer, offering a more scalable and cost-effective approach to remote monitoring. The **E4 set** consisted of the raw, minimally preprocessed biosignals available directly from the E4 (ACC, ACC vector magnitude, BVP, HR, EDA, and skin temperature), highlighting the device’s out-of-the-box predictive potential. The **ACC/EDA set** featured the two biosignals that have consistently shown strong performance for seizure detection in previous research [31]. Finally, the **Full set** incorporated all available biosignals, the derived phasic and tonic components of EDA, sleep–wake states from ACC, and frequency-domain features of all biosignals. In addition, each feature set included the static features age, sex, and device placement (left or right wrist). A complete breakdown of the features included in each set is provided in Supplementary Materials, Section S2.

Model performance was evaluated using three key metrics: Area under the receiver operating characteristic curve (AUROC), seizure-wise recall (SW-Recall), and false alarms per hour (FA/h). SW-Recall, a modified form of traditional recall, measures performance at the seizure-event level rather than the segment level [7,32]. Under this metric, a seizure is counted as a true positive if *any* segment within the seizure is correctly classified as ictal. If no segments are correctly identified, the seizure is marked as a false negative. This approach aligned with our primary objective of evaluating whether a model could detect the occurrence of a seizure, rather than the proportion of correctly detected segments within a seizure. In contrast, FA/h quantifies the average number of false positives the model produces per hour, offering a practical measure of clinical usability by estimating how often a device might incorrectly signal a seizure.

## 3. Results

### 3.1. Patient Population

The study included 28 patients with epilepsy. The median age was 24.5 years (IQR, [17, 33.25]) and 10 (36%) were women. A total of 25 (89%) patients identified as White, 1 (4%) as American Indian/Alaskan Native, and 2 (7%) did not report their race. A total of 26 (93%) patients identified as not Hispanic or Latino, with 2 (7%) identifying as Hispanic or Latino. The median number of seizures experienced by patients while under observation was 3 (IQR, [2, 4.25]), with a median duration of 113 (IQR, [92, 158]) seconds. A total of 17 (61%) participants experienced a single type of seizure, while 11 (39%) participants experienced multiple seizure types. The full demographic details are provided in Table 1, stratified by seizure type.

### 3.2. Seizure Detection Performance

Our experiments assessed various ML model types, feature sets, and segment lengths to determine whether specific approaches performed best for certain seizure types and whether a single approach may be optimal for *all* seizure types. From across all tested combinations of ML model types, hyperparameters, feature sets, and segment lengths, the top ten best-performing models within each seizure type are presented for comparison, with performance ranked by AUROC.

#### 3.2.1. Seizure Types

**Figure 1:**
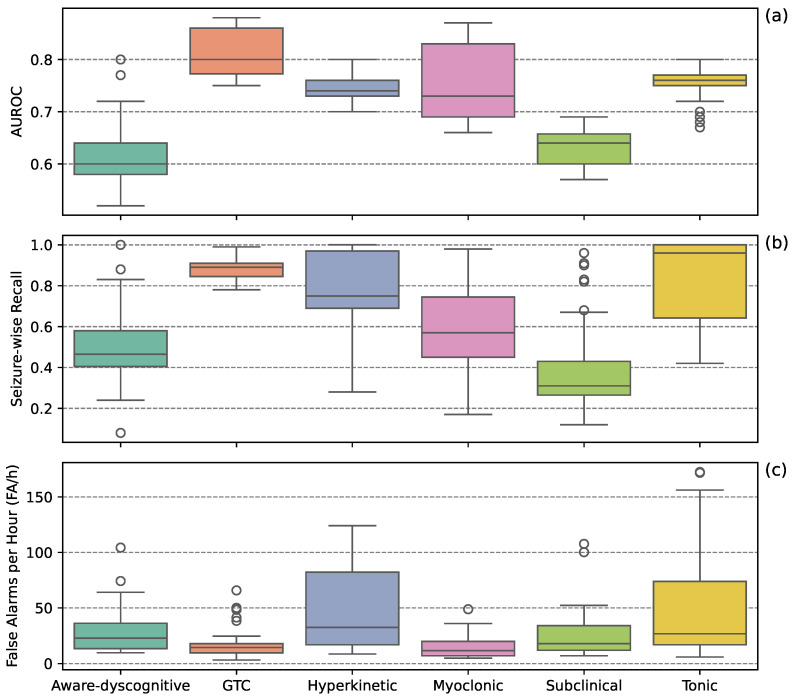
Detection performance across seizure types. Boxplots show AUROC (**a**), seizure-wise recall (**b**), and false alarms per hour (**c**) for the top 10 models per seizure type.

Figure 1 presents boxplots representing the distribution of individual performance scores from the top ten models for each seizure type. GTC seizures had the highest AUROC scores while maintaining the lowest FA/h. Hyperkinetic and tonic seizures showed high SW-Recall but also the highest FA/h. Myoclonic seizures demonstrated modest SW-Recall with low FA/h. Aware-dyscognitive and subclinical seizures exhibited the lowest SW-Recall and high FA/h.

#### 3.2.2. Machine Learning Model Types

**Figure 2:**
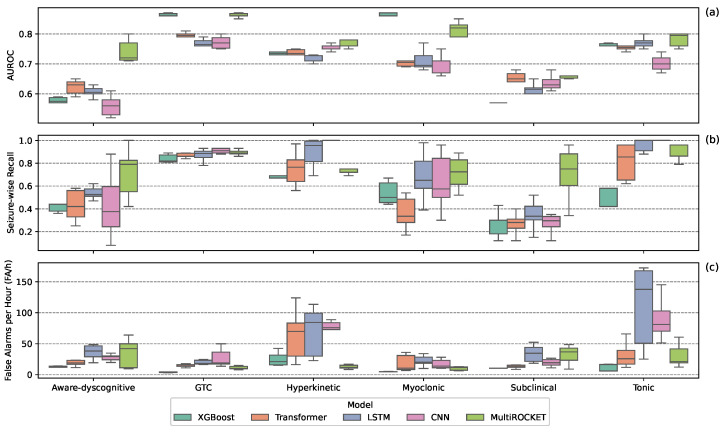
Detection performance across seizure types, separated by machine learning model type. Boxplots show AUROC (**a**), seizure-wise recall (**b**), and false alarms per hour (**c**) for the top 10 models per seizure type.

Figure 2 presents boxplots comparing model performance across seizure types. MultiROCKET performed well for aware-dyscognitive seizures, balancing SW-Recall and FA/h. GTC seizures were the best classified overall, with XGBoost and MultiROCKET achieving high SW-Recall with low FA/h. Hyperkinetic seizures were challenging, with most models showing high FA/h; MultiROCKET was the exception, maintaining high SW-Recall and low FA/h. For myoclonic seizures, XGBoost showed modest SW-Recall and low FA/h, while LSTMs, CNNs, and MultiROCKET demonstrated higher SW-Recall at the cost of higher FA/h. Subclinical seizures were the hardest to classify, with low AUROC and SW-Recall across models; although MultiROCKET led in SW-Recall, this came with higher FA/h. Subclinical seizures were hardest to classify, with low AUROC and SW-Recall across models; although MultiROCKET led in SW-Recall, it also had higher FA/h. Tonic seizures were similarly difficult, with generally high FA/h and low SW-Recall; XGBoost achieved modest SW-Recall and lower FA/h. Considering overall performance, while MultiROCKET, on average, achieved the highest AUROC scores, no single model consistently outperformed the rest.

#### 3.2.3. Feature Sets

**Figure 3:**
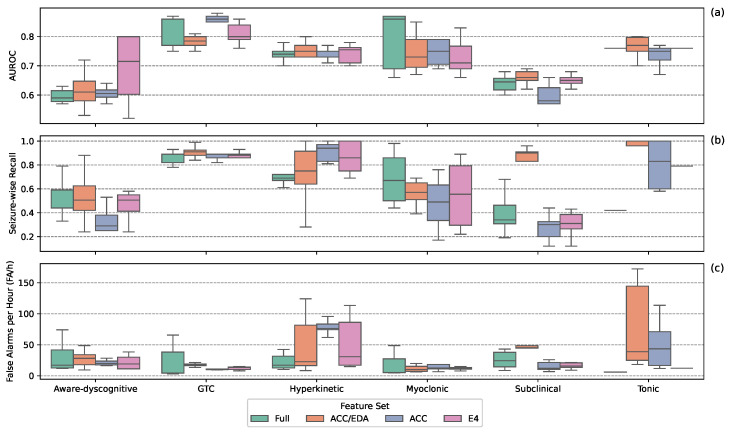
Detection performance across seizure types, separated by feature set. Boxplots show AUROC (**a**), seizure-wise recall (**b**), and false alarms per hour (**c**) for the top 10 models per seizure type.

Figure 3 shows performance by feature set across seizure types. For aware-dyscognitive seizures, all sets except the ACC set, had modest SW-Recall and FA/h; with the ACC set showing lower SW-Recall. GTC seizures were consistently well detected, with high AUROC and low FA/h across all sets. Hyperkinetic seizures remained difficult: all sets achieved high SW-Recall, but most suffered from high FA/h, except the Full set. Myoclonic seizure performance varied, with low FA/h across sets; the Full set had the highest SW-Recall, while the ACC set had the lowest. Subclinical seizures were challenging for all models; most sets had low SW-Recall and FA/h, though the ACC/EDA set showed high SW-Recall with high FA/h. For tonic seizures, the E4 set achieved high SW-Recall and low FA/h, while others showed trade-offs between these metrics indicative of poor performance. Overall, feature set effectiveness varied by seizure type, with no set consistently outperforming the others.

#### 3.2.4. Segment Lengths

**Figure 4:**
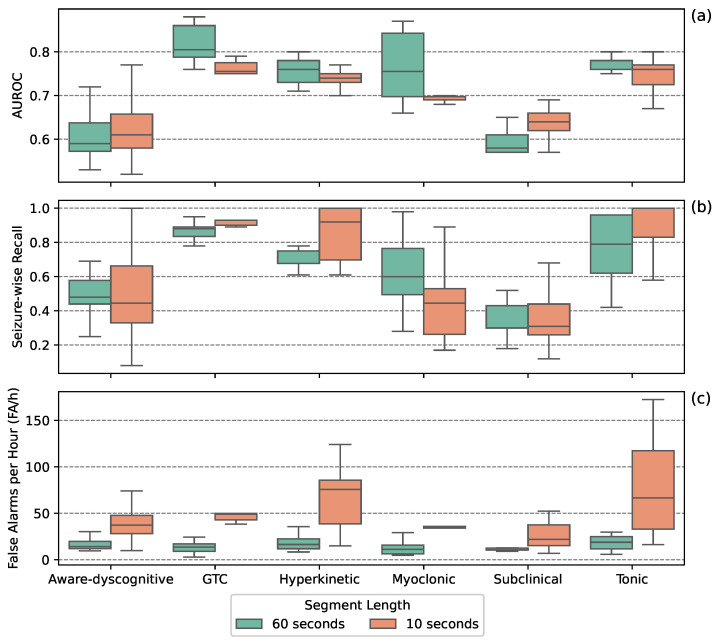
Detection performance across seizure types, separated by segment length. Boxplots show AUROC (**a**), seizure-wise recall (**b**), and false alarms per hour (**c**) for the top 10 models per seizure type.

Figure 4 illustrates detection performance when stratified by segment length. In general, longer segments (60 s) yielded better results, significantly reducing FA/h across most seizure types while maintaining competitive SW-Recall. Shorter segments (10 s) provided higher SW-Recall for GTC, hyperkinetic, and tonic seizures, but at the cost of significantly higher FA/h.

#### 3.2.5. Best-Performing Approach

The best-performing approach varied by seizure type. Referring to Table 2, for GTC seizures, XGBoost with the Full set and 60 s segments achieved the highest overall performance (AUROC = 0.86; SW-Recall = 0.81; FA/h = 3.03). Myoclonic seizures were also best detected by XGBoost using the Full set and 60 s segments (AUROC = 0.86; SW-Recall = 0.50; FA/h = 4.79). Hyperkinetic seizures were best classified by MultiROCKET with the ACC/EDA set and 60 s segments, but encountered a high FA/h (AUROC = 0.76; SW-Recall = 0.75; FA/h = 8.44). For tonic seizures, XGBoost with the Full set and 60 s segments performed best (AUROC = 0.76; SW-Recall = 0.42; FA/h = 5.79). Aware-dyscognitive seizures were optimally detected by MultiROCKET using the ACC/EDA set and 60 s segments, with a notably high FA/h (AUROC = 0.72; SW-Recall = 0.50; FA/h = 9.56). Finally, subclinical seizures, the most difficult to detect, were best classified by XGBoost with the ACC set and 10 s segments (AUROC = 0.57; SW-Recall = 0.12; FA/h = 6.89). Participant-level results for the best-performing models are provided in Table 3.

## 4. Discussion

### 4.1. Principal Findings

This study evaluated the feasibility of using wrist-worn wearables and ML methods to detect seizures, extending previous research beyond the commonly studied GTC [7]. Our results align with previous work, demonstrating reliable GTC detection across all model types and feature sets [32,33]. Non-motor seizures (aware-dyscognitive and subclinical) remained challenging to detect, with high variability in SW-Recall and FA/h across models, also consistent with earlier studies [34]. These results support the hypothesis that seizure semiologies vary between patients, suggesting patient-specific models may improve performance [35]. Our study evaluated a variety of ML models, feature sets, and segment lengths, finding that specific approaches may be better for certain seizure types.

#### 4.1.1. Machine Learning Model Types

XGBoost and MultiROCKET exhibited distinct strengths: XGBoost performed best for GTC and myoclonic seizures, while MultiROCKET excelled in detecting aware-dyscognitive, hyperkinetic, and tonic seizures. XGBoost was particularly effective for seizures with strong movement patterns, whereas MultiROCKET was better suited for subtle, variable seizure types. MultiROCKET’s superior performance for aware-dyscognitive and hyperkinetic seizures highlights the value of rich temporal feature extraction for these seizure types. Future work should consider a hybrid approach: using XGBoost on features extracted from MultiROCKET, which may enhance classification across more seizure types.

#### 4.1.2. Feature Selection

Feature selection played a significant role in detection performance, with multi-biosignal feature sets enhancing classification accuracy across seizure types. The Full set exhibited, on average, the lowest FA/h across all seizure types, although sometimes at the cost of lower SW-Recall. The ACC/EDA set, on average, did not excel for any particular seizure category. While prior work has shown the combination of ACC and EDA improves GTC detection [36], these results suggest the incorporation of additional biosignals can further enhance performance. The ACC set, while competitive for motor seizures, performed poorly for aware-dyscognitive and subclinical seizures, supporting previous findings that ACC alone is insufficient for non-motor seizures [7]. Despite generally low AUROC for aware-dyscognitive seizures, the E4 set performed relatively well, balancing SW-Recall and FA/h. This may reflect reduced overfitting, as the Full set’s high dimensionality may hinder generalization. No feature set proved universally superior, underscoring the need for seizure-specific biosignal combinations. Future work should explore adaptive feature selection tailored to patient-specific seizure patterns to improve detection accuracy.

#### 4.1.3. Segment Length

Longer segment lengths (60 s) generally reduced FA/h, though this may partly be due to their reduced prediction frequency, as 60 s models made predictions every minute, where as 10 s models made predictions every 10 s. Nevertheless, 60 s models showed improved accuracy for GTC, hyperkinetic, myoclonic, and tonic seizures, while remaining competitive for aware-dyscognitive and subclinical seizures. These findings align with prior work suggesting that seizures may exhibit stronger predictive physiological signatures over extended temporal windows [37].

### 4.2. Comparison with Prior Work

Previous studies combining wearable devices with ML have primarily focused on detecting GTC seizures. While these studies have often demonstrated strong performance, they have generally been limited in the range of seizure types considered. More recently, however, this scope has expanded with researchers investigating a broader spectrum of seizure types. Two prominent works include Tang et al., who trained ML models on sensor data from both the wrist and ankle to detect nine different seizure types [7], while Yu et al. similarly, applied deep learning models to wrist and ankle sensor data for detecting 28 seizure types in a pediatric population [38].

Placing our results within the context of these previous studies, we observed reasonably consistent detection performance for multiple seizure types. Across all three studies, GTC seizures were detected with the highest accuracy: our best-performing model achieved an AUROC of 0.86, compared to 0.98 reported by Tang et al. [7], and 0.97 by Yu et al. [38]. For aware-dyscognitive seizures (referred to as “focal behavior arrest” in both prior studies), our model achieved an AUROC of 0.72, closely aligning with Tang et al’s 0.71 and lower than Yu et al’s 0.81. Yu et al. reported strong performance for hyperkinetic seizures (AUROC 0.95) and myoclonic seizures (AUROC 0.74), compared to our AUROC of 0.76 for hyperkinetic and 0.86 for myoclonic seizures. A direct match for these seizure types was unavailable in Tang et al’s work. Subclinical seizures proved particularly challenging across all studies, with modest detection performance: our AUROC of 0.57 was comparable to Tang’s 0.55 and lower than Yu’s 0.67. Lastly, detection of tonic seizures showed moderate to strong performance, with our model achieving an AUROC of 0.76, compared to 0.81 in Tang et al. and 0.79 in Yu et al.

The observed variations in performance scores may be attributed to differences in sensor placement (e.g., inclusion of ankle-worn devices), population characteristics, dataset size, and training or validation methodologies. Despite these differences, our findings were largely consistent with these recent studies, reaffirming that while GTC seizures can be reliably detected using wearable devices, the identification of more subtle focal and subclinical seizures remains a significant challenge. Our study complements these prior works by evaluating a broad range of ML models, including XGBoost and ROCKET. Notably, for non-GTC seizures, models such as MultiROCKET and XGBoost often outperformed individual deep learning models in our study, suggesting that hand-crafted and time-series features can complement deep learning approaches.

### 4.3. Potential for Accelerometry-Only Devices

Previous research has shown promise in using ACC-only devices for seizure detection [8,39]. A systematic review by Sasseville et al. reported that ACC devices achieved sensitivity rates of ≥80% and FA/h of ≤1 per day for GTC seizures [40]. Our findings support this, as the ACC feature set performed well for motor seizures, particularly GTC, where movement is a defining characteristic. However, ACC alone underperformed for non-motor seizures compared to multi-biosignal feature sets, highlighting the value of integrating additional biosignals to improve detection across seizure types.

Despite their limitations, ACC-based models remain attractive for resource-limited settings given their lower cost [41]. While patients have reported satisfaction with wearables for at-home seizure detection, concerns about affordability may hinder widespread adoption [42]. A survey by Quiroga et al. found that among 92 respondents, only 6.6% reported they would use such a device regardless of cost [43]. Therefore, optimizing ACC-based models could address these barriers by providing an accessible option for both low-resource healthcare settings and widespread clinical use [42,44].

### 4.4. Impact of False Alarms on Clinical Implementation

In addition to cost, high false alarm rates are a significant barrier to adoption in at-home and clinical settings. Frequent false positives can erode user trust and hinder device acceptance [42,43]. A systematic review by Hadady et al. reported that patients generally expect a minimum sensitivity of 90%, with acceptable false alarm rates varying from 1 to 2 per week to 1 to 2 per month, emphasizing that failure to meet these expectations may result in device abandonment [42]. In our study, some ML models met sensitivity expectations, but false alarms, particularly for hyperkinetic and tonic seizures, remain a major limitation. While current performance may still hold clinical value for medication management [45], future work must prioritize reducing false alarms without compromising sensitivity.

### 4.5. Limitations

This study has several limitations. First, all experiments were conducted using data from a single cohort at one center, limiting the generalizability and reproducibility of our findings. The lack of multicenter data raises questions about how the models would perform in different clinical settings, which may include more diverse seizure presentations and monitoring protocols. Second, the cohort consisted predominantly of adults, and the findings may not generalize to pediatric or geriatric populations. Third, the sample size for certain seizure types, particularly hyperkinetic and tonic seizures, was relatively small, which may have impacted model performance and stability. Finally, external validations on larger, independent datasets is essential to confirm the robustness and clinical applicability of these models.

## 5. Conclusions

This study highlights the potential of wrist-worn wearables combined with ML to extend seizure detection beyond GTC seizures. By evaluating multiple models, feature sets, and segment lengths, we underscore the importance of methodological optimization for achieving reliable detection across seizure types. Our findings demonstrate strong performance for motor seizures (e.g., GTC, hyperkinetic), with relatively high seizure-wise recall and manageable false alarm rates. In contrast, non-motor seizures (e.g., aware-dyscognitive, subclinical) proved more difficult to detect, with lower recall and higher false alarm rates. These differences point to the need for seizure-specific approaches and signal combinations. Future work should focus on minimizing false alarms, integrating multimodal biosignals, and developing personalized, adaptive models to improve accuracy and clinical utility. Addressing these challenges will be pivotal for integrating wearable-based seizure detection systems into routine clinical practice, enabling real-time remote monitoring and improving outcomes for patients with epilepsy.

## Figures and Tables

**Table 1:** Subject demographics.

	Full Cohort	Aware-Dyscognitive	Generalized Tonic–Clonic	Hyperkinetic	Myoclonic	Subclinical	Tonic
**N Patients (N Seizures)**	28 (109)	6 (16)	14 (25)	4 (12)	6 (16)	8 (28)	6 (12)
**Sociodemographic variables**							
Age: median (IQR)	24.5 (17, 33.25)	18.5 (17.25, 25)	31 (18.75, 38.5)	37 (27.25, 49.75)	42 (28, 54.5)	17.5 (14.75, 35.5)	19.5 (15, 21)
Sex, N (%)							
Female	10 (36)	3 (50)	5 (36)	1 (25)	1 (17)	6 (75)	1 (17)
Male	18 (64)	3 (50)	9 (64)	3 (75)	5 (83)	2 (25)	5 (83)
Race, N (%)							
American Indian / Alaskan Native	1 (4)	0 (0)	1 (7)	0 (0)	0 (0)	1 (12)	0 (0)
White	25 (89)	5 (83)	12 (86)	4 (100)	6 (100)	6 (75)	6 (100)
Not reported	2 (7)	1 (17)	1 (7)	0 (0)	0 (0)	1 (12)	0 (0)
Ethnicity, N (%)							
Hispanic or Latino	2 (7)	0 (0)	0 (0)	1 (25)	0 (0)	0 (0)	1 (17)
Not Hispanic or Latino	26 (93)	6 (100)	14 (100)	3 (75)	6 (100)	8 (100)	5 (83)
**Clinical variables**							
Number of seizures per patient: median (IQR)	3 (2, 4.25)	2 (1.25, 3.5)	1.5 (1, 2)	1 (1, 3)	1 (1, 2.5)	1.5 (1, 4.5)	1 (1, 3.25)
Seizure duration, in seconds: median (IQR)	113 (92, 158)	113 (83, 130)	130 (102, 154)	136 (103, 240)	142 (101, 230)	100 (83, 212)	98 (90, 106)

**Table 2:** Best-performing model for each seizure type.

Seizure Type	Model	Feature Set	Segment Length	AUROC	Seizure-Wise Recall	False Alarms per Hour (FA/h)
Aware-dyscognitive	Multirocket	ACC/EDA	60 s	0.72	0.50	9.56
Generalized tonic–clonic	XGBoost	Full	60 s	0.86	0.81	3.03
Hyperkinetic	Multirocket	ACC/EDA	60 s	0.76	0.75	8.44
Myoclonic	XGBoost	Full	60 s	0.86	0.50	4.79
Subclinical	XGBoost	ACC	10 s	0.57	0.12	6.89
Tonic	XGBoost	Full	60 s	0.76	0.42	5.79

**Table 3:** Best-performing model for each seizure type: Participant-level results.

Seizure Type	Model	Feature Set	Sequence Length	Participant	AUROC	Seizure-Wise Recall	False Alarms per Hour (FA/h)
Aware-dyscognitive	MultiROCKET	ACC/EDA	60 s	0	0.79	1.0	18.89
				1	0.54	0.0	6.02
				2	0.78	0.5	4.06
				3	0.93	1.0	6.83
				4	0.64	0.0	8.74
				5	0.62	0.5	12.83
GTC	XGB	Full	60 s	0	0.95	1.0	2.67
				1	0.88	1.0	2.24
				6	0.96	1.0	5.31
				7	0.87	1.0	0.78
				8	1.0	1.0	6.62
				9	0.93	0.8	8.6
				10	1.0	1.0	3.2
				11	1.0	1.0	3.27
				12	0.66	0.0	2.5
				13	0.61	1.0	0.91
				14	1.0	1.0	0.86
				15	0.84	0.0	2.89
				16	0.91	0.5	2.31
				17	0.38	1.0	0.3
Hyperkinetic	MultiROCKET	ACC/EDA	60 s	18	0.66	1.0	18.03
				19	1.0	1.0	0.76
				20	0.46	0.0	6.41
				21	0.91	1.0	8.56
Myoclonic	XGB	Full	60 s	0	0.66	0.0	9.5
				10	0.84	0.0	1.57
				11	0.93	0.0	3.39
				18	0.89	1.0	3.89
				22	0.88	1.0	4.65
				23	1.0	1.0	5.76
Subclinical	XGB	ACC	10 s	4	0.5	0.0	4.37
				5	0.46	0.0	9.26
				7	0.56	0.0	0.05
				8	0.29	0.0	8.31
				10	0.58	0.0	5.16
				15	0.9	0.0	3.98
				17	0.52	1.0	22.56
				24	0.72	0.0	1.39
Tonic	XGB	Full	60 s	0	0.56	0.0	10.56
				1	0.79	0.0	9.23
				4	0.93	1.0	8.61
				25	0.75	1.0	2.6
				26	0.73	0.0	1.74
				27	0.77	0.5	2.01

## Data Availability

The datasets generated during and/or analyzed during the current study are not publicly available because participant's raw Empatica and seizure log data contain protected health information and are subject to HIPAA regulations. As such, they cannot be shared publicly to ensure patient privacy and confidentiality. However, special arrangements can be made for qualified researchers trained in human subjects confidentiality protocols to securely assess the data set and program code used in this study behind the Mayo Clinic firewall. Researchers interested in accessing the data may contact irbservicecenter@mayo.edu to discuss potential data access under appropriate data use agreements and institutional review board (IRB) approval.

## Supplementary Materials

The following supporting information can be downloaded at: https://www.mdpi.com/article/10.3390/s25175562/s1, Section S1: Model Hyperparameters. Section S2 Feature Sets.

## Abbreviations

ACC	Accelerometry
BVP	Blood volume pulse
PPG	Photoplethysmography
EDA	Electrodermal activity
HR	Heart rate
EEG	Electroencephalograph
vEEG	Video electroencephalogram
GTC	Generalized tonic–clonic
LSTM	Long Short-Term Memory
CNN	Convolutional Neural Network
XGBoost	Extreme Gradient Boosting
ROCKET	Random Convolution Kernel Transform
SW-Recall	Seizure-wise recall
IQR	Interquartile range
ML	Machine learning
