# Supplementary material for: Detecting Diverse Seizure Types with Wrist-Worn Wearable Devices: A Comparison of Machine Learning Approaches
Source: Sensors (Basel). 2025 Sep 6;25(17):5562. doi: 10.3390/s25175562 (PMC12431448; doi:10.3390/s25175562)
Supplement: Supplementary file 1 [file sensors-25-05562-s001.zip › sensors-3722714-supplementary.pdf]

S1 Model Hyperparameters

1. **XGB**
   (a) Number of estimators: 50, 100, 500
   (b) Maximum depth: 3, 10, 30, 50
   (c) Learning rate: 0.01, 0.1, 0.2
   (d) Subsample: 0.8
2. **LSTM**
   (a) Training epochs: 50, 200
   (b) Learning rate: 0.001
   (c) Dropout: 0.2
   (d) Hidden size: 32, 64, 128
   (e) Number of layers: 2, 4
3. **CNN**
   (a) Training epochs: 50, 200
   (b) Learning rate: 0.001
   (c) Dropout: 0.2
   (d) Number of filters: [32, 64, 128]
   (e) Kernel sizes: [4, 4, 4], [8, 8, 8]
   (f) Pooling sizes: [2, 2, 2], [4, 4, 4]
4. **Transformer**
   (a) Training epochs: 50, 200
   (b) Learning rate: 0.001
   (c) Dropout: 0.2
   (d) Hidden size: 32, 64, 128
   (e) Number of layers: 2, 4
   (f) Number of heads: 2, 4
5. **ROCKET variations**
   (a) ROCKET
      i. Number of kernels: 10,000, 30,000
   (b) Mini-ROCKET
      i. Number of kernels: 10,000, 30,000
      ii. Maximum dilations per kernel: 32
   (c) Multi-ROCKET
      i. Number of kernels: 10,000, 30,000
      ii. Maximum dilations per kernel: 32
      iii. Number of features per kernel: 4

S2 Feature Sets

Table S1: Overview of variables and signals included in each feature set.

	**Feature Sets**			
	**ACC**	**ACC/EDA**	**E4**	**Full**
**Variable**				
Age	✓	✓	✓	✓
Sex	✓	✓	✓	✓
Wrist Placement (Right or Left)	✓	✓	✓	✓
Time of Day (Hour)	✓	✓	✓	✓
**Signal**				
Accelerometry, X-axis	✓		✓	✓
Accelerometry, Y-axis	✓		✓	✓
Accelerometry, Z-axis	✓		✓	✓
Accelerometry, Vector Magnitude	✓	✓	✓	✓
Blood Volume Pulse			✓	✓
Electrodermal Activity, Raw		✓	✓	✓
Electrodermal Activity, Phasic				✓
Electrodermal Activity, Tonic				✓
Skin Temperature			✓	✓
Heart Rate			✓	✓
Rest/Wake State				✓
Peak Frequency*				✓
Spectral Entropy*				✓
Spectral Centroid*				✓
Spectral Bandwidth*				✓
Total Power*				✓

* Computed for each signal.
